# Diffusion Tensor Tractography Shows White Matter Tract Changes in Breast Cancer Survivors with Balance Impairment

**DOI:** 10.3390/pathophysiology32040063

**Published:** 2025-11-19

**Authors:** Alexandra Nikolaeva, Maria Pospelova, Mark Voynov, Varvara Krasnikova, Albina Makhanova, Samvel Tonyan, Aleksandr Efimtsev, Fionik Olga, Anatoliy Levchuk, Gennadiy Trufanov, Konstantin Samochernykh, Tatyana Alekseeva, Stephanie E. Combs, Maxim Shevtsov

**Affiliations:** 1 Personalized Medicine Centre, Almazov National Medical Research Centre, Akkuratova Str. 2, 197341 Saint Petersburg, Russia; pospelovaml@mail.ru (M.P.); m.vojnov@mail.ru (M.V.); krasnikova_vv@almazovcentre.ru (V.K.); a.mahanova.a@mail.ru (A.M.); samvelium@gmail.com (S.T.); atralf@mail.ru (A.E.); fvolga@mail.ru (F.O.); feuerlag999@yandex.ru (A.L.); trufanovge@mail.ru (G.T.); neurobaby12@gmail.com (K.S.); t.alekseeva@mail.ru (T.A.); 2 Laboratory of Biomedical Nanotechnologies, Institute of Cytology of the Russian Academy of Sciences (RAS), Tikhoretsky Ave., 4, 194064 Saint Petersburg, Russia; stephanie.combs@tum.de; 3 Department of Radiation Oncology, Technishe Universität München (TUM), Klinikum Rechts der Isar, Ismaninger Str. 22, 81675 Munich, Germany

**Keywords:** breast cancer, breast cancer survivors, “chemobrain”, diffusion tensor imaging, magnetic resonance imaging, tractography

## Abstract

**Objectives**: Breast cancer survivors often experience long-term neurological complications, including balance impairments, following treatment. This study aimed to investigate microstructural changes in white matter tracts in breast cancer survivors with balance impairment using diffusion tensor tractography. **Methods**: An open, single-center, prospective study was conducted including two groups—healthy age-matched volunteers (*n* = 28) and breast cancer survivors (*n* = 35) with balance impairment. All participants underwent diffusion tensor tractography at baseline and at the end of the follow-up period of six months. Quantitative anisotropy was analyzed using DSI Studio to assess white matter integrity. **Results**: At baseline, patients with balance impairment exhibited significantly reduced quantitative anisotropy values in the middle cerebellar peduncles (*p* = 0.046) and cerebellar hemispheres (*p* = 0.024, 0.055) compared to healthy controls. At the end of the follow-up, quantitative anisotropy values were increased across most tracts, though some differences persisted between groups (*p* < 0.001). **Conclusions**: Breast cancer survivors with balance impairment demonstrate sustained microstructural white matter changes, particularly in cerebellar and vestibular pathways. These findings suggest that diffusion tensor tractography can provide valuable insights into central nervous system alterations contributing to post-treatment balance dysfunction and may serve as a potential tool for early diagnosis and rehabilitation planning.

## 1. Introduction

Cancer remains one of the leading causes of morbidity and mortality worldwide [1]. In most countries in Europe, North and South America, it ranks as the first or second most common cause of death among adults under 70 years of age [2]. The most prevalent malignancies include lung, breast, colorectal and prostate cancers [3]. Breast cancer (BC) alone represents the second most frequently diagnosed cancer; according to GLOBOCAN, in 2022, more than 2.3 million new cases and over 660,000 deaths were reported globally [3]. Due to advances in medicine, BC is now detected at earlier stages, and the proportion of patients with metastatic breast cancer at the time of diagnosis has significantly decreased in recent years [4]. Substantial progress has also been achieved in treatment: surgical and reconstructive techniques have improved, and adjuvant and neoadjuvant chemotherapy, targeted therapy, endocrine therapy and immunotherapy are increasingly effective [5,6,7].

Despite the availability of these therapies and the resulting decrease in mortality, most women who have undergone treatment for breast cancer experience a range of persistent problems that substantially impair their life quality [8]. The literature often addresses specific complications experienced by breast cancer survivors under separate terms such as “post-mastectomy pain syndrome” [9,10], “chemotherapy-related cognitive impairment” [11] or chemotherapy-induced peripheral neuropathy (CIPN) [12,13]. Neurological manifestations span central, peripheral and autonomic nervous system dysfunctions [14].

Particular attention has recently been paid to central nervous system (CNS) alterations in breast cancer survivors. Such changes result from the combined effects of surgery, chemotherapy and radiotherapy, and include cognitive, emotional and balance disturbances [15,16,17,18]. Cognitive-emotional disorders may occur in 20–50% of patients after breast cancer treatment [19]. Anxiety symptoms typically include excessive worry, fear of recurrence, muscle tension and irritability [20]; sleep onset and quality disturbances are also frequent, especially among patients with lymphedema [21]. Depression affects approximately 30% of this population [15,22], with frequent complaints of hopelessness, loss of interest, fatigue, guilt and worthlessness [20]. Cognitive impairments include short-term memory loss, difficulties with learning new information, concentration and word-finding problems and a “brain fog” sensation [23]. The phenomenon of chemotherapy-induced cognitive impairment (“chemobrain”) has been extensively documented [24]. Cytotoxic agents including alkylating agents, microtubule destabilizers, stabilizers and antimetabolites have all been shown to impair neurogenesis, myelination and neurotransmitter regulation, induce neuroinflammation and trigger neuronal apoptosis [25]. The underlying mechanisms include reduced neuro- and gliogenesis, dendritic atrophy, neuroinflammation and oxidative stress, resulting in loss of gray and white matter and hippocampal atrophy [26]. Several studies emphasize microstructural white-matter changes as a key factor in long-term cognitive decline after breast cancer treatment [27].

Balance impairment in breast cancer survivors is characterized by dizziness, gait instability and syncope and has been described in detail previously [28]. According to Prayuenyong P. et al., approximately 17% of cancer survivors treated with cisplatin experience balance disturbances such as dizziness or unsteadiness, which are significantly associated with peripheral neuropathy symptoms [29]. There is also substantial evidence regarding cisplatin, a widely used chemotherapeutic agent, which is well known for its ototoxic effects primarily resulting from damage to cochlear hair cells, often leading to hearing loss, tinnitus, and dizziness in treated patients [30]. Nevertheless, the central mechanisms underlying balance impairment in breast cancer survivors have been largely overlooked in existing studies.

Magnetic resonance imaging (MRI) is a promising modality to assess alterations in white matter tracts in breast cancer survivors. A wide range of MRI techniques is available, from standard pulse sequences (T1, T2, DWI, SWI) to advanced methods such as MR angiography, diffusion and perfusion imaging, functional MRI, spectroscopy and contrast-enhanced studies [31]. Each has distinct advantages, limitations and clinical applications. In our previous study, using voxel-based morphometry (VBM) on MRI, we identified a statistically significant reduction in both gray and white matter volumes in breast cancer survivors [32].

In our current study, we chose to explore the method of diffusion tensor tractography (DTI tractography) in breast cancer survivors with balance impairment, which may enhance our understanding of the pathomorphological and pathophysiological changes of the CNS in the long-term period following breast cancer treatment. DTI has already been applied in studies of breast cancer survivors to investigate chemotherapy-related cognitive impairment [33] and depression [34], but, to date, no studies have explored its use in cohorts of patients with balance impairment.

The aim of the present study was to assess changes in white matter tracts in breast cancer survivors using DTI tractography at two time points—at baseline and again after a 6-month follow-up period—in order to evaluate microstructural alterations and their progression over time. An open, single-center, prospective study was conducted involving two groups: healthy volunteers (control group) and breast cancer survivors with balance impairment (main group). In this study, balance impairment was defined as the presence of subjective complaints of dizziness, unsteadiness, or gait instability, confirmed by abnormalities during standard neurological examination. Objective assessment included the Romberg test, coordination tests, and the Tinetti Performance-Oriented Mobility Assessment. Patients demonstrating deviations in one or more of these tests were classified as having balance impairment. Herein, we hypothesize that breast cancer survivors will show significant alterations in the integrity of white matter tracts compared with healthy volunteers. We further hypothesize that these changes will persist at follow-up, potentially relating to the vestibular symptoms observed after breast cancer treatment.

## 2. Materials and Methods

This open, single-center, non-controlled study was conducted to investigate alterations in white matter tracts of the brain using DTI tractography in women in the long-term period following breast cancer treatment. The study was carried out in accordance with the principles of the Declaration of Helsinki of the World Medical Association and was approved by the Ethics Committee of the Almazov National Medical Research Centre, Ministry of Health of the Russian Federation (Extract No. 050112-2022 from the meeting No. 0112-22 12 December 2022). Written informed consent was obtained from all participants prior to enrollment.

### 2.1. Patients and Healthy Volunteers

A total of 35 female patients with a confirmed diagnosis of stage II or III breast cancer who had completed a course of chemotherapy between 6 and 12 months prior to enrollment were included in the study. The mean age of participants ranged from 35 to 44 years (43.2 ± 4.30 years) in the late postoperative period (>2 years) with a mean time since surgery of 3.35 ± 1.59 years. Additional inclusion criteria included right-handedness. Of the participants, 23 women had undergone Madden mastectomy, 7 underwent sectoral resection, 5 underwent subcutaneous mastectomy and 12 underwent mastectomy with immediate mammoplasty (Table 1). Chemotherapy regimens varied: FAC was administered to 2 patients, CAF to 3 patients, docetaxel/paclitaxel to 27 patients, and AC to 13 patients. Hormone therapy was received by 23 patients, and radiotherapy by 29 patients. The control group of healthy volunteers included age-matched women, with a mean age of 44 ± 5.68 years (range, 28–46 years).

Exclusion Criteria: progression of the main oncological disease; presence of distant metastases (including brain metastases); diseases of the brain and cerebral vessels (such as brain tumors, demyelinating diseases, developmental anomalies, traumatic brain injury, aneurysms, arteriovenous malformations, hemodynamically significant stenoses of the head and neck main arteries, and other relevant pathologies); decompensated somatic pathology; acute infectious and mental diseases; pregnancy; contraindications to MRI.

All the study participants underwent a neurological examination, which included the Romberg test, coordination tests, and Tinetti scale testing, complaints and medical history were collected. Balance impairment was verified based on positive findings in at least one of the following: Romberg test, coordination tests, or Tinetti scale. Based on these results, 47 patients were excluded from the study due to manifestations of damage to the peripheral nervous system (poly- and mononeuropathies, a history of systemic dizziness). The remaining 35 patients had no concomitant somatic pathology, signs of progression of the underlying cancer, contraindications to MRI, uncorrectable decrease in visual acuity, signs of oto- and vestibulotoxicity, polyneuropathy, cerebrovascular pathology, lymphedema of the 2nd or more stage, severe scoliotic deformity, asthenic syndrome. The average BMI of the participants was 26.4 ± 4.2 kg/m^2^. Approximately 40% of the participants reported taking medications, including antihypertensives and pain relievers. Comorbidities were present in 35% of the sample, most commonly hypertension and diabetes, all of which were well-compensated.

### 2.2. MRI Study

The study was performed using a 3-Tesla Magnetom Vida Siemens MRI scanner (Siemens Healthineers AG, Forchheim, Germany). All patients underwent DTI tractography. MRI scans were performed between 6 and 12 months after completion of chemotherapy (baseline point), and repeated after a 6-month follow-up period. Thus, the total time since the end of therapy to the second MRI ranged from 12 to 18 months. Diffusion tensor MRI scanning was performed with a b-value of 1000 s/mm^2^ and 20 diffusion-encoding directions. Motion correction was applied during post-processing using DSI Studio software (version 2021). Eddy current correction and geometric distortion correction were not applied in this study. The pulse sequence parameters are presented in Table 2.

### 2.3. Therapy of Balance Disorders

For 6 months, all patients received non-specific treatment: patients from the comparison group (without clinical manifestations of balance disorders)- comprehensive physical rehabilitation, patients of the main group (with verified imbalance)- neurometabolic therapy (capsules of ethylmethylhydroxypyridine succinate 250 mg × 3 times a day for 6 months, sachets of citicoline 1000 mg 1 time a day)- 2 months, as well as comprehensive physical rehabilitation, including aerobic exercise: moderate-intensity cardio training under the guidance of a physical therapy instructor for 40 min 3 times a week.

### 2.4. Statistical Analysis

Data quality was controlled via visual inspection in DSI Studio, with removal of geometric distortions and motion-related artifacts. A brain mask was then generated to suppress background signal, improve reconstruction efficiency, and streamline subsequent visualization. All remaining DTI preprocessing steps were executed automatically [35].

Statistical analyses were performed using Statistica 12.5 software (TIBCO Software Inc., Palo Alto, CA, USA). The normality of distributions for each structure was assessed using the D’Agostino–Pearson test with visual inspection of the plots. For normally distributed data, results were presented as mean (SD) and compared between two independent groups using a two-tailed *t*-test with α = 0.05. For non-normal data, results were presented as median (IQR) and compared using the Mann–Whitney test. For multiple comparisons across anatomical structures, the Holm–Bonferroni correction (FWER) was applied; tables present adjusted *p*-values (p_adj), with significance defined as p_adj < 0.05.

Postprocessing of diffusion tensor imaging data and group comparisons were performed using DSI Studio. Quantitative anisotropy (QA) was extracted as the local connectome fingerprint for connectometry analysis. QA was selected as the primary metric for analysis, as it more accurately reflects the directional flow of water molecules along neural fibers and is less affected by free water or edema compared with fractional anisotropy. QA, derived from generalized q-sampling imaging (GQI), is linked to axonal density and is considered more sensitive to axonal loss than fractional anisotropy, as it is less influenced by edema. A two-step filtering strategy was applied. Fibers with QA below a predefined threshold were removed to eliminate noise and define tract endpoints, followed by orientation selection in voxels with multiple directions, favoring the smallest turning angle. Propagation was then calculated. Group connectometry (correlational tractography) was conducted to assess QA in white matter tracts of patients with post-breast cancer treatment syndromes (chronic pain, vestibulocerebellar ataxia, depression). The syndrome state served as a variable, and nonparametric Spearman correlation was used for analysis, with whole-brain set as the default region.

A T-score threshold of 2.5 guided deterministic fiber tracking. QA values were normalized, and tracks were refined using four iterations of topology-informed pruning. An FDR threshold of 0.05 with 4000 random permutations was applied to control false positives. The analysis identified tracts showing both positive and negative correlations with the clinical variable. Of particular interest were tracts with reduced QA, reflecting decreased axonal integrity in patients after treatment or with specific syndromes compared to controls or unaffected patients.

## 3. Results

All participants—healthy volunteers (control group) and breast cancer survivors with balance impairment (main group)—underwent DTI tractography.

Patients with balance disorders underwent MR tractography within 6 months. During this time, the study participants received complex non-specific therapy to reduce the severity of balance disorders and improve overall well-being.

Table 3 presents the results of quantitative anisotropy measurements obtained using diffusion tensor imaging (DTI) and tractography in patients who had undergone breast cancer treatment (main group) and in healthy volunteers at the first study point. Comparative analysis demonstrated statistically significant reductions in QA values in several cerebellar structures among patients compared to healthy controls. Specifically, the middle cerebellar peduncles (*p* = 0.046) and the left cerebellar hemisphere (*p* = 0.024) showed significant differences. No significant differences were found in the cerebellar vermis, right inferior cerebellar peduncle, cranial nerve VIII, or right brainstem–reticular tract, indicating that these regions maintained relatively preserved diffusion characteristics at this stage of observation.

**Figure 1:**
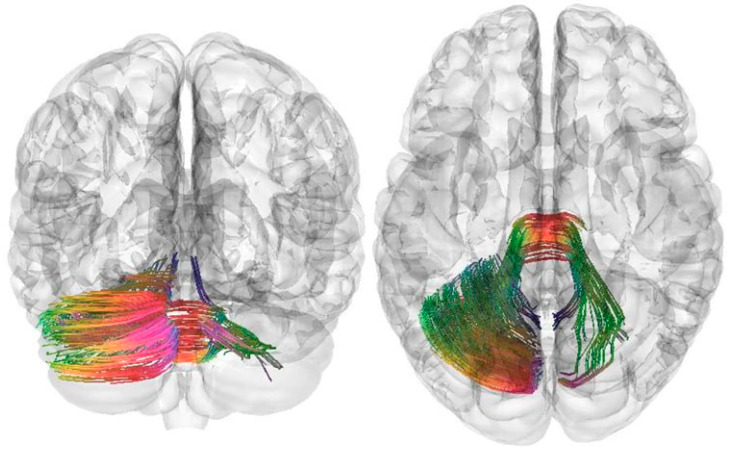
Three-dimensional reconstruction of the white matter tracts of the left cerebellar hemisphere and middle cerebellar peduncles in female patients with balance disorders compared to a group of healthy volunteers. Red indicates the fiber direction along the X-axis (right-left), green indicates the Y-axis (front-back), and blue indicates the Z-axis (up-down).

Tractography is a method for reconstructing white brain fibers based on the analysis of diffusion-weighted MRI data, which makes it possible to quantify the microstructure of white matter through parameters such as fractional anisotropy (FA). When comparing two groups of patients, the data underwent standardized preprocessing, pathway reconstruction using deterministic or probabilistic tractography algorithms, after which quantitative white matter metrics were extracted for statistical analysis of differences. DSI Studio supported methods that provide greater accuracy and reliability when analyzing patient groups. This approach is widely used to identify changes in white matter associated with pathology or treatment effects, and allowed us to identify significant neuroanatomical features between groups with high statistical reliability. Figure 1 illustrates the white matter tracts that demonstrated statistically significant differences in quantitative anisotropy between breast cancer survivors with balance impairment and healthy controls. Only the tracts showing significant group differences are visualized, primarily involving the middle cerebellar peduncles and the left cerebellar hemisphere. The color selection in the drawing was determined automatically by the program and depended on the location of the fibers along the x, y, and z axes; blue indicates movement from top to bottom, red—from left to right, and green—from front to back (Figure 1) [36,37,38,39].

At Point 2, a comparison between the main group and healthy volunteers revealed statistically significant differences in the quantitative anisotropy of the middle cerebellar peduncles (*p* = 0.016) and cranial nerve VIII (*p* = 0.013). The results are presented in Table 4.

In the final comparison of patients from the main group between Point 1 and Point 2, a statistically significant increase in quantitative anisotropy was observed at Point 2 across all examined structures, with the exception of the right brainstem–reticular tract (Table 5).

## 4. Discussion

Dizziness and balance impairment are frequent yet underrecognized complications following chemotherapy in patients with different types of cancer [40]. Despite their significant impact on quality of life and functional recovery, these symptoms are often underestimated in clinical practice and remain insufficiently explored in current research. Most studies have focused predominantly on peripheral mechanisms contributing to balance impairment, particularly those associated with chemotherapy-induced peripheral polyneuropathy [41,42,43]. In contrast, central mechanisms—specifically the structural and functional alterations of the brain—have received comparatively little attention, despite growing neuroimaging evidence suggesting CNS involvement in post-treatment neurological syndromes.

In the present study, we aimed to elucidate the pathophysiological and pathomorphological correlates of CNS involvement in breast cancer survivors with balance impairment using DTI tractography. The investigation targeted cerebellar tracts and the vestibulocochlear nerve (cranial nerve VIII), as these structures play a crucial role in balance regulation, movement coordination, and spatial orientation. At the first study point, breast cancer survivors with balance impairment exhibited significantly reduced quantitative anisotropy in several cerebellar structures compared with healthy volunteers. The middle cerebellar peduncles and the left cerebellar hemisphere demonstrated the most pronounced changes, suggesting early microstructural alterations of white matter integrity that may reflect treatment-related neurotoxicity and demyelination. By the second study point, an increase in QA was observed across the examined structures in patients with balance impairment relative to the first point. This pattern may reflect compensatory neuroplasticity within central nervous system pathways, highlighting adaptive mechanisms that contribute to the maintenance or recovery of postural control despite earlier microstructural disruption. Similar observations have been reported in studies of Parkinson’s disease. In particular, Jilu Princy Mole et al. found that fractional anisotropy in the motor tracts of Parkinson’s patients was increased relative to healthy controls, and they proposed that such an increase might reflect compensatory reorganization of neural circuits—i.e., adaptive neuroplasticity rather than solely degeneration [44]. Although this pattern could potentially reflect compensatory neuroplasticity within central nervous system pathways, this interpretation remains hypothesis-generating.

The observed findings align with the hypothesis that vestibular dysfunction in breast cancer survivors can result not only from peripheral injury but also from central maladaptation. The potential involvement of the inner ear, particularly the degeneration of vestibular hair cells, provides a plausible peripheral substrate for the symptoms observed [45]. Structural degeneration in vestibular pathways corresponds closely with clinical manifestations of dizziness, gait instability, and impaired spatial orientation in these patients [46]. Platinum-based chemotherapeutic agents—cisplatin, carboplatin, and oxaliplatin—are known to induce vestibulotoxicity through apoptosis of sensory hair cells, excessive oxidative stress, mitochondrial dysfunction, and the release of proinflammatory cytokines [30]. The primary pathological processes involve hair cell apoptosis, oxidative stress, and the release of proinflammatory cytokines, all of which contribute to hair cell degeneration and subsequent disruption of vestibular function [30]. These insights underscore the complex interplay between peripheral vestibular damage and central nervous system adaptations, providing a mechanistic basis for the observed post-treatment balance deficits and highlighting the importance of integrating both peripheral and central perspectives in the study of chemotherapy-related neurotoxicity. Cerebellar involvement is consistent with prior observations in neurodegenerative conditions. For instance, Prakash N. et al. reported comparable microstructural abnormalities, specifically demonstrating reductions in fractional anisotropy within the cerebellar peduncles of patients with spinocerebellar ataxia [47]. The concordance between those observations and our findings lends weight to the interpretation that lowered diffusion anisotropy in cerebellar tracts signals compromised white-matter integrity—most plausibly reflecting axonal degeneration, myelin disruption, and increased extracellular water associated with neurodegenerative or injury-related processes [44].

Several limitations of this study should be acknowledged. The single-center design and relatively small sample size may limit the generalizability of our findings. The lack of longitudinal clinical balance assessments parallel to MRI data restricts our ability to directly correlate microstructural recovery with functional improvement. Additionally, although chemotherapy regimens were recorded, their heterogeneity and cumulative doses were not analyzed in relation to the observed imaging changes, which could provide further mechanistic insight. The relatively small sample size and the heterogeneity of chemotherapy regimens, which may have distinct neurotoxic profiles, represent important limitations of this study and may restrict the generalizability of the findings. Future studies should include larger, multicenter cohorts, detailed clinical-vestibular evaluations, and multimodal imaging approaches—such as functional MRI and MR spectroscopy—to comprehensively assess both structural and functional aspects of CNS alterations in this population.

Collectively, these findings highlight a dual-phase trajectory of CNS alterations in breast cancer survivors: an initial phase of vulnerability characterized by microstructural loss and decreased anisotropy, followed by a potential recovery phase that may reflect compensatory neuroplastic changes. This dynamic pattern supports the hypothesis that the brain retains a capacity for structural adaptation even after systemic oncologic treatment and its neurotoxic effects, although the neuroplasticity interpretation remains tentative. Recognizing such potential plasticity is crucial for designing targeted rehabilitation interventions aimed at promoting recovery of balance and coordination

## 5. Conclusions

Our findings demonstrate that breast cancer survivors with balance impairment exhibit persistent microstructural alterations in cerebellar and vestibular white matter tracts, even months after completing therapy. These changes likely reflect central mechanisms underlying post-treatment balance disturbances. Diffusion tensor tractography thus emerges as a sensitive, non-invasive method for detecting subtle CNS alterations and may contribute to early identification and targeted rehabilitation of neurological complications in breast cancer survivors.

## Figures and Tables

**Table 1:** Surgical Treatment Tactics.

	Main Group	
	Abs.	(%)
Madden Mastectomy	23	65.7
Sectoral Mastectomy	7	20.0
Subcutaneous Mastectomy	5	14.3
Mastectomy with Immediate Breast Reconstruction	12	34.3
Side of Intervention: left	13	37.1
Side of Intervention: right	22	62.9
Total	35	100

**Table 2:** Pulse sequence parameters of DTI.

DTI Parameters	Pulse Sequence
Repetition time/TR	2800.0 ms
Echo time/TE	79.00 ms
FoV	220 mm
Slice thickness	3.0 mm
Flip angle	90°
Voxel size x (mm), y (mm)	1.7 × 1.7 × 3.0 mm
Study time	3:37

**Table 3:** Results of QA in patients of the main group and healthy volunteers at Point 1.

Structure	Healthy Volunteers	Main Group	*p*-Value
Middle cerebellar peduncles	0.191 ± 0.063	0.175 ± 0.053	0.046
Cerebellar vermis	0.125 ± 0.035	0.130 ± 0.043	0.510
Right cerebellar hemisphere	0.149 ± 0.045	0.127 ± 0.036	0.055
Left cerebellar hemisphere	0.172 ± 0.054	0.139 ± 0.039	0.024
Right inferior cerebellar peduncle	0.165 ± 0.056	0.171 ± 0.057	0.421
Cranial nerve VIII	0.189 ± 0.078	0.188 ± 0.055	0.396
Right brainstem–reticular tract	0.190 ± 0.091	0.195 ± 0.048	0.784

**Table 4:** Results of QA in patients of the main group and healthy volunteers at Point 2.

Structure	Main Group	Healthy Volunteers	*p*-Value
Middle cerebellar peduncles	0.257 ± 0.064	0.191 ± 0.063	0.016
Cerebellar vermis	0.164 ± 0.038	0.125 ± 0.035	0.054
Right cerebellar hemisphere	0.176 ± 0.051	0.149 ± 0.045	0.095
Left cerebellar hemisphere	0.169 ± 0.028	0.172 ± 0.054	0.948
Right inferior cerebellar peduncle	0.219 ± 0.056	0.165 ± 0.056	0.152
Cranial nerve VIII	0.274 ± 0.081	0.189 ± 0.078	0.013
Right brainstem–reticular tract	0.274 ± 0.026	0.190 ± 0.091	0.055

**Table 5:** Results of QA in patients of the main group at Point 1 and Point 2.

Structure	Main Group, Point 1	Main Group, Point 2	*p*-Value
Middle cerebellar peduncles	0.175 ± 0.053	0.257 ± 0.064	0.001
Cerebellar vermis	0.130 ± 0.043	0.164 ± 0.038	0.021
Right cerebellar hemisphere	0.127 ± 0.036	0.176 ± 0.051	0.001
Left cerebellar hemisphere	0.139 ± 0.039	0.169 ± 0.028	0.011
Right inferior cerebellar peduncle	0.171 ± 0.057	0.219 ± 0.056	0.009
Cranial nerve VIII	0.188 ± 0.055	0.274 ± 0.081	0.001
Right brainstem–reticular tract	0.195 ± 0.048	0.274 ± 0.026	0.095

## Data Availability

The datasets used and/or analyzed during the current study are available from the corresponding authors Alexandra Nikolaeva and Maxim Shevtsov on reasonable request.

## Abbreviations

The following abbreviations are used in this manuscript:

CNS	Central Nervous System
DTI tractography	Diffusion Tensor Tractography
DWI	Diffusion-Weighted Imaging
MRI	Magnetic Resonance Imaging
QA	Quantitative Anisotropy
WM	White Matter
